# Naturally occurring SARS-CoV-2 gene deletions close to the spike S1/S2 cleavage site in the viral quasispecies of COVID19 patients

**DOI:** 10.1080/22221751.2020.1806735

**Published:** 2020-09-02

**Authors:** Cristina Andrés, Damir Garcia-Cehic, Josep Gregori, Maria Piñana, Francisco Rodriguez-Frias, Mercedes Guerrero-Murillo, Juliana Esperalba, Ariadna Rando, Lidia Goterris, Maria Gema Codina, Susanna Quer, Maria Carmen Martín, Magda Campins, Ricard Ferrer, Benito Almirante, Juan Ignacio Esteban, Tomás Pumarola, Andrés Antón, Josep Quer

**Affiliations:** a Respiratory Viruses Unit, Microbiology Department, Vall d’Hebron Institut de Recerca (VHIR), Vall d’Hebron Hospital Universitari, Barcelona, Spain; b Liver Unit, Liver Diseases - Viral Hepatitis, Vall d’Hebron Institut de Recerca (VHIR), Vall d’Hebron Hospital Universitari, Barcelona, Spain; c Centro de Investigación Biomédica en Red de Enfermedades Hepáticas y Digestivas (CIBERehd), Instituto de Salud Carlos III, Madrid, Spain; d Roche Diagnostics SL, Sant Cugat del Valles, Barcelona, Spain; e Biochemistry and Microbiology Departments, Vall d’Hebron Institut de Recerca (VHIR), Vall d’Hebron Hospital Universitari, Barcelona, Spain; f Universitat Autònoma de Barcelona, Bellaterra, Spain; g Microbiology Department, Vall d’Hebron Hospital Universitari, Barcelona, Spain; h Preventive Medicine, Hospital Vall d’Hebron Hospital Universitari, Barcelona, Spain; i Intensive Care Department. Shock, Disfunció Orgànica i Ressuscitació (SODIR) Research Group, Vall d’Hebron Institut de Recerca (VHIR), Vall d’Hebron Hospital Universitari, Barcelona, Spain; j Infectious Diseases Department. Vall d’Hebron Institut de Recerca (VHIR), Vall d’Hebron Hospital Universitari, Barcelona, Spain

**Keywords:** SARS-CoV-2, deletions, quasispecies, NGS, respiratory virus, diversity

## Abstract

The SARS-CoV-2 spike (S) protein, the viral mediator for binding and entry into the host cell, has sparked great interest as a target for vaccine development and treatments with neutralizing antibodies. Initial data suggest that the virus has low mutation rates, but its large genome could facilitate recombination, insertions, and deletions, as has been described in other coronaviruses. Here, we deep-sequenced the complete SARS-CoV-2 *S* gene from 18 patients (10 with mild and 8 with severe COVID-19), and found that the virus accumulates deletions upstream and very close to the S1/S2 cleavage site (PRRAR/S), generating a frameshift with appearance of a stop codon. These deletions were found in a small percentage of the viral quasispecies (2.2%) in samples from all the mild and only half the severe COVID-19 patients. Our results suggest that the virus may generate free S1 protein released to the circulation. We suggest that natural selection has favoured a “Don’t burn down the house” strategy, in which free S1 protein may compete with viral particles for the ACE2 receptor, thus reducing the severity of the infection and tissue damage without losing transmission capability.

## Introduction

RNA viruses replicate using their own RNA-dependent RNA polymerase (RdRp), which lacks proofreading mechanisms and is prone to mutate at high rates (10^−3^–10^−5^ substitutions/nucleotide/replication cycle), lending the virus a quasispecies structure [1,2]. Previous studies with severe acute respiratory syndrome coronavirus (SARS-CoV) and mouse hepatitis virus have reported moderate mutation rates of 9.06 × 10^−7^ and 2.5 × 10^−6^ subs/site/cycle respectively, below the expected range for RNA viruses [3]. This is consistent with a role for non-structural protein (nsp) 14 in RNA proofreading or repair functions because of its 3’-5’ exonuclease (ExoN) activity. Nonetheless, the large size of the CoV RNA genome increases the probability that deletions will be generated and recombination events will take place, which could facilitate adaptation to new host environments, as occurs with jumping between species[1,2]*.* One naturally occurring deletion on 29 nucleotides in the open reading frame (ORF) 8 of SARS-CoV after human-to-human transmission was found to be associated with attenuation of replication [4].

The low mutation rate, high human-to-human transmissibility (*R*_0_ = 2.2) [5], and absence of human pre-existing immunity against SARS-CoV-2 could explain its rapid spread through the human population, with very high sequence identity (99.9%) between isolates recovered all over the world (sequence published in the repository sequence data banks, GISAID and GenBank). The high pathogenicity of the virus, the severity of coronavirus disease-19 (COVID-19) and the lack of an effective antiviral treatment or vaccine has pushed the scientific community worldwide to develop, in record time, a solution for this pandemic [6].

Among the SARS-CoV-2 structural proteins, including spike (S), envelope (E), and membrane (M) constituting the viral coat, and the nucleocapsid (N) protein that packages the viral genome, the S glycoprotein is the most promising as a therapeutic and vaccine target. The S protein is encoded by the *S* gene, and following trimerization, it composes the spikes of the characteristic viral particle crown (corona). The S protein is essential for SARS-CoV-2 to infect a host cell [7] by recognizing and binding to the human cell receptor, angiotensin-converting enzyme 2 (ACE2) [8], and possibly (with lower affinity) to other receptors, such as CD209L (L-SIGN), also used by SARS-CoV [9] and dipeptidyl peptidase 4 (DPP4), used by MERS [10].

The *S* gene has 3822 nucleotides with 1273 amino acids (GenBank reference sequence MN908947.3). It has five essential domains: the receptor-binding domain (RBD), O-linked glycan residues flanking a polybasic S1/S2 cleavage site, fusion peptide (FP), heptad repeats HR1 and HR2, and a transmembrane domain (TM). The S1 RBD includes 6 amino acid positions that show high affinity for the human ACE2 receptor, which is widely distributed, but mainly present in alveolar type 2 (AT2) cells of the lungs [11]. Once the virus is attached to the host cell receptor, cleavage occurs between subunits S1 and S2, and subunit S2 drives the viral and cellular membranes to fuse [12]. Thus, S1 recognizes and binds to the human cell receptor ACE2, whereas S2 directly facilitates entry into the host cell. Both functions are crucial for infection, and therein lies the interest of S as a target for the development of vaccines and antiviral agents.

Because of the importance of the S protein, we carried out a deep-sequencing study of the *S* gene in upper respiratory tract samples from 18 patients with mild or severe SARS-CoV-2 disease. Of particular note, hot-spot deletion sites were found in minority mutants located upstream and very close to the S1/S2 (PRRAR/S) and S2’ (KPSKR/SFI) cleavage sites, suggesting that these genomes code for a truncated S protein. The variants were significantly more prevalent in patients with mild than those with severe disease. Thus, their effect on the protein could constitute a favourable regulatory mechanism emerging in the viral quasispecies to modulate the pathological effect of the infection. Discussion is provided on the implications this observation may have in the biology of SARS-CoV-2.

## Patients and methods

### Patients

Upper respiratory tract specimens (naso/oropharyngeal swabs or nasopharyngeal aspirates) from individuals consulting in the emergency room were collected for SARS-CoV-2 testing in the Department of Microbiology at Hospital Universitari Vall d’Hebron (HUVH), Barcelona (Spain). Samples from 18 patients with no previous comorbidities other than COVID-19 were included in the study. As defined by CDC criteria (https://www.cdc.gov/coronavirus/2019-ncov/hcp/clinical-guidance-management-patients.html), 10 patients had a mild clinical presentation of COVID-19 (absence of viral pneumonia and hypoxia, no hospitalization requirement, able to manage their illness at home), whereas 8 patients had severe disease (intensive care unit (ICU) admission for supportive management of complications of severe COVID-19 such as pneumonia, pneumonia, hypoxemic respiratory failure, sepsis, cardiomyopathy and arrhythmia, acute kidney injury, and other complications). All patients were attended by March 2020, and those with both mild and severe disease had a favourable outcome with resolution of the infection.

## Methods

### Detection of SARS-CoV-2

The diagnosis of COVID-19 was performed by two tests, an in-house RT-PCR assay using the primer/probe set from the CDC 2019-nCoV Real-Time RT–PCR Diagnostic Panel (Qiagen, Hilden, Germany) and a commercial real-time RT-PCR assay (Allplex 2019-nCoV Assay, Seegene, South Korea).

### SARS-CoV-2 sequencing

The 18 respiratory specimens were inactivated by mixing 140 µL of the sample with 560 µL of AVL buffer (Qiagen, Hilden, Germany). Extraction of nucleic acids was then performed using the QIAmp Viral RNA Mini Kit (Qiagen, Hilden, Germany) following the manufacturers’ instructions but without the RNA carrier, obtaining a final elution of 30 µL.

The complete *S* gene was amplified using a double PCR. The first RT–PCR step consisted in amplifying 2 large fragments, 3314 base pairs (bp) and 3591 bp in length, respectively. The 5’ end of primer 1 and 3’ end of primer 2 were designed to be outside the *S* region to ensure that we were amplifying SARS-CoV-2 genomic RNA, and not subgenomic RNA (Table S11).

The SuperScript III One-Step RT–PCR System with Platinum Taq HiFi DNA Polymerase (Invitrogen; Carlsbad, CA, USA) was used for the RT–PCR. Reverse transcription was done at 50°C for 30 min, followed by a retrotranscriptase inactivation step at 94°C for 2 min. Next, 30 cycles of PCR amplification were performed as follows: denaturation at 94°C for 15 sec, annealing at 54°C for 30 sec, and elongation at 68°C for 5 min. After the last cycle, amplification ended with a final elongation step at 68°C for 5 min.

The second round of amplification (nested) was done using overlapping internal primer pairs to amplify fragments 470 bp to 313 bp in length. The FastStart High-Fidelity PCR System dNTPack (Sigma, St. Louis, MO, CA) was used for this purpose, as follows: activation at 94°C for 4 min, followed by 30 cycles with denaturation at 94°C for 30 sec, annealing at 55°C for 30 sec, and elongation at 72°C for 40 sec, ending with a single elongation step at 72°C for 7 min.

PCR products were purified using the QIAquick Gel Extraction Kit (Qiagen, Hilden, Germany) with QG buffer, following the manufacturers’ instructions, and eluted DNA was quantified by fluorometry using the QUBIT dsDNA BR Assay Kit (ThermoFisher, MA, USA). For each patient, PCR products were normalized to 1.5 ng/µL, pooled in a single tube, and purified using KAPA Pure Beads (KapaBiosystems, Roche, Pleasanton, CA, USA) to ensure that no short DNA fragments were present in the library. Library preparation was done using the KAPA Hyper Prep Kit (Roche Applied Science, Pleasanton, CA, USA) and each pool was individually indexed using the SeqCap Adapter Kit A/B (Nimblegen, Roche, Pleasanton, CA, USA). After library enrichment and a second clean-up with KAPA Pure Beads, the pools were quantified again using the QUBIT dsDNA BR Assay Kit and quality-tested using the 4150 TapeStation System (Agilent, Santa Clara, CA, USA). All pools underwent a final normalization to 4 nM, and 10 µL of each pool was added to the final library tube. The final library was qPCR-quantified using the KAPA Library Quantification Kit (KapaBiosystems, Roche, Pleasanton, CA USA) in a LightCycler 480 system (Roche) to obtain the precise concentration of indexed DNA. PhiX V3 internal DNA control (Illumina, San Diego, CA, USA) was added to the final dilution. The library was loaded in a MiSeq Reagent Kit 600V3 cartridge (Illumina, San Diego, CA) and sequenced using the MiSeq platform (Illumina, San Diego, CA).

### Bioinformatics analysis. InDel study

The sequence analysis aimed to obtain high-quality haplotypes fully covering the amplicons. The pipeline comprises the following steps:

1. Amplicons were reconstructed from the corresponding R1 and R2 paired ends using FLASH [13] and setting a minimum of 20 overlapping bases and a maximum of 10% mismatches. Low-quality reads that did not meet the requirements were discarded.
2. Next, all reads with more than 5% of bases below a Phred score of Q30 were filtered out.
3. The reads were demultiplexed by matching primers, allowing a maximum of three mismatches, and the primers were trimmed at both read ends. Identical reads were collapsed to haplotypes with the corresponding frequencies as read counts. A fasta file was generated with each pool/primer/strand combination. The reverse haplotypes were reverse complemented.
4. Raw forward and reverse haplotypes were multiple aligned with MUltiple Sequence Comparison by Log-Expectation (MUSCLE) [14], then separated into strands, and haplotypes common to both strands at abundances ≥0.1% were identified. Low-abundance haplotypes (<0.1%) and those unique to one strand were discarded. The haplotypes common to both strands, with frequencies not below 0.1% were called consensus haplotypes, and were the basis of subsequent computations.

The amino acid alignments were computed as follows:

1. Gaps were removed and haplotypes translated to amino acids.
2. The translated stops generated were identified, and haplotypes were trimmed after the stop.
3. Resulting amino acid haplotypes were realigned with MUSCLE (EMBL-EBI https://www.ebi.ac.uk/Tools/msa/muscle/).

All computations were made in the R language and platform [15], developing in-house scripts using Biostrings [16] and Ape [17] packages.

## Results

Eighteen COVID-19 patients (10 mild and 8 severe) were included in the study. In total, 48,746,647 reads, ranging from 81,202 to 597,558 reads per amplicon (median 171,478), were analysed from upper respiratory tract samples (Table 1), using 13 overlapping amplicons covering the complete S protein. Thus, we studied 3,749,742 complete *S* genes, with a mean of 208,319 per patient. Sequences have been uploaded to the GenBank Sequence Read Archive (SRA) database with BioProject accession number PRJNA630679. Results related to amplicon positions, coverage, percentage of the master sequence, gap incidence per patient and per amplicon, and premature stop codons are available as Supplementary Tables S1–S10.

**Table 1.** Characteristics of patients with mild and severe COVID-19. #P16 had clinical symptoms consistent with severe disease, but he was not hospitalized in the ICU.

Sample Id (*P* = patient)	Real-time PCR cycle threshold (Ct) value	COVID-19 classification	Sample Type	Sex (F = female; M = male)	Age (years)	Days at Intensive Care Unit (ICU)
P01	19.00	Mild	nasopharyngeal aspirate	F	34	no admission
P02	25.00	mild	nasopharyngeal aspirate	F	54	no admission
P03	16.40	mild	nasopharyngeal/oropharyngeal swab	F	42	no admission
P04	23.10	mild	nasopharyngeal/oropharyngeal swab	F	25	no admission
P05	25.98	mild	nasopharyngeal/oropharyngeal swab	M	52	no admission
P06	21.45	mild	nasopharyngeal/oropharyngeal swab	F	42	no admission
P07	25.94	mild	nasopharyngeal/oropharyngeal swab	F	25	no admission
P14	23.71	mild	nasopharyngeal aspirate	F	26	no admission
P15	27.32	mild	nasopharyngeal/oropharyngeal swab	M	41	no admission
P18	15.50	mild	nasopharyngeal/oropharyngeal swab	M	74	no admission
P08	No data	severe	nasopharyngeal/oropharyngeal swab	F	51	4
P09	25.36	severe	nasopharyngeal/oropharyngeal swab	M	49	3
P10	21.23	severe	nasopharyngeal/oropharyngeal swab	F	47	16
P11	36.01	severe	nasopharyngeal/oropharyngeal swab	M	45	27
P12	31.04	severe	nasopharyngeal/oropharyngeal swab	M	51	23
P13	22.94	severe	nasopharyngeal aspirate	F	44	55
P16	34.35	severe	nasopharyngeal/oropharyngeal swab	F	45	#
P17	30.77	severe	nasopharyngeal/oropharyngeal swab	M	49	10

Deletions were not randomly accumulated along the *S* gene, but instead, were found at specific regions (Figure 1, Figures S1–S27). Deletions coded as delta (Δ1–Δ18) ranged from 1 to 42 nucleotides lost (Table 2). In some cases, the sequence recovered the correct reading frame, in others, the frameshift caused the appearance of a premature stop codon very close to the deletion site, whereas in still others, a new amino acid segment appeared.

**Figure 1.**
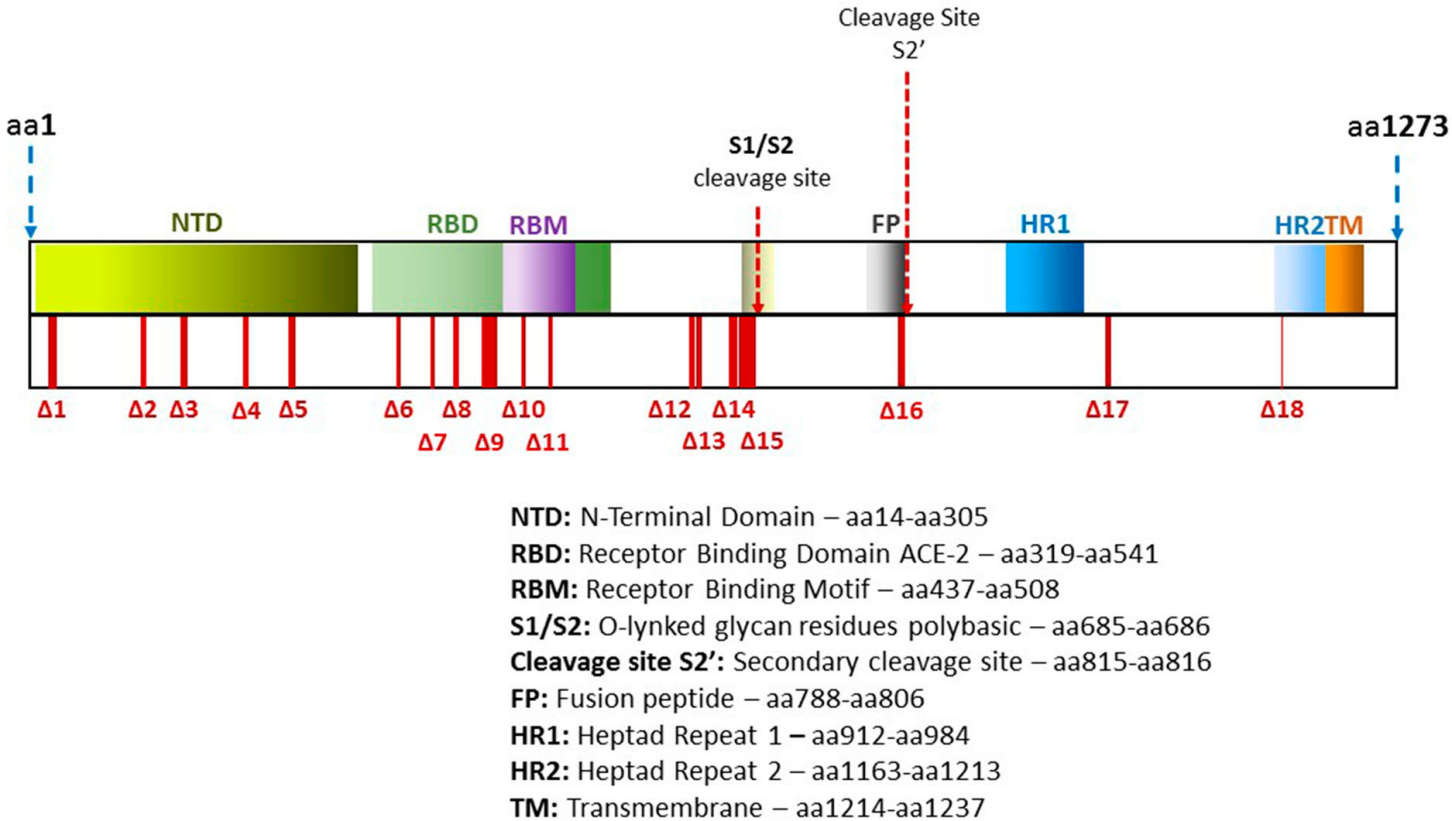
Diagram showing location of the deletions found along the Spike gene and protein^29^.

**Table 2.** List of deletions found along the spike gene.

Deletion code (Δ=deletion region)	Amplicon at the nucleotide level	Deleted nucleotide positions	Deleted amino acid positions	Number of nts deleted	Patient code	Number of reads with deletions	Total reads	Population frequency (in percentage)
Δ1	N01	38–49	13S-17V	12	**P01**	234	126,140	0.19
Δ2	N01	323–329	108T-110L	2–7	**P01-P04-P05-P09-914**	7329	648,955	1.13
Δ3	N02	420–434	140F-145Y	2–14	**P01-P02-P04-P05-P06-P09**	10,111	1,617,589	0.63
Δ4	N02	596–603	199G-201F	2–8	**P02-P04-P09**	1031	653,783	0.16
Δ5	N03	724–736	242L-246R	5–6	**P02-P09**	2024	259,012	0.78
Δ6	N03-N04	1022–1027	341V-343N	2–4	**P01-P02-P04-P06**	3809	1,042,526	0.37
Δ7	N04	1120–1128	374F-S-396T	9	**P06**	387	157,405	0.25
Δ8	N04	1177–1180	393T-394N	4	**P01**	338	155,414	0.22
Δ9	N04-N05	1283–1324	428D-442D	13–42	**P04-P09**	3835	496,156	0.77
Δ10	N05	1368–1376	456F-459S	9	**P01**	245	172,903	0.14
Δ11	N06	1444–1452	482G-484E	9	**P01**	521	173,278	0.30
Δ12	N07	1865–1870	622V-A-624I	6	**P09-P17**	16,725	436,054	3.84
Δ13	N07	1888–1894	630T-*P*-632T	19	**P04**	193	148,533	0.13
Δ14	N07	1961–1979	654E-660Y	8	**P09**	1068	192,954	0.55
Δ15	N07	1980–2035	660Y-679N	2–34	**P01-P02-P03-P04-P05-P06-P07-P08-P09-P10-P11-P14-P15-P18**	64,978	2,923,548	2.22
Δ16	N08-N09	2451–2467	817F-823F	2–16	**P01-P03-P04-P05-P06-P09-P14-P15-P18**	11,739	2,176,059	0.54
Δ17	N10-N11	3018–3019	1006T-1007Y	2	**P01**	1544	299,893	0.51
Δ18	N12-N13	3499	1167G	1	**P08**	23,359	472,695	4.94

Deletions were found in all amplicons, but they were mainly observed at frequencies <1% (Table 2). Most deletions in amplicons N04, N05, N06, N10, N11, N12, and N13, were found in only 1 or 2 patients, whereas deletions in amplicons N01, N02, N08 and N09, ranging from 2 to 16 nucleotides, were observed in 4–9 patients. A deletion of 6 nucleotides in amplicon N07 (nt 1865–1870), generating a stop codon, was present at a frequency of 3.84% of the quasispecies in samples from patients P09 and P17. The largest deletion, involving 42 nucleotides (nt 1283–1324) and found in N05 of patient P09, resulted in a loss of 14 amino acids, but the reading frame recovered.

**Figure 2.**
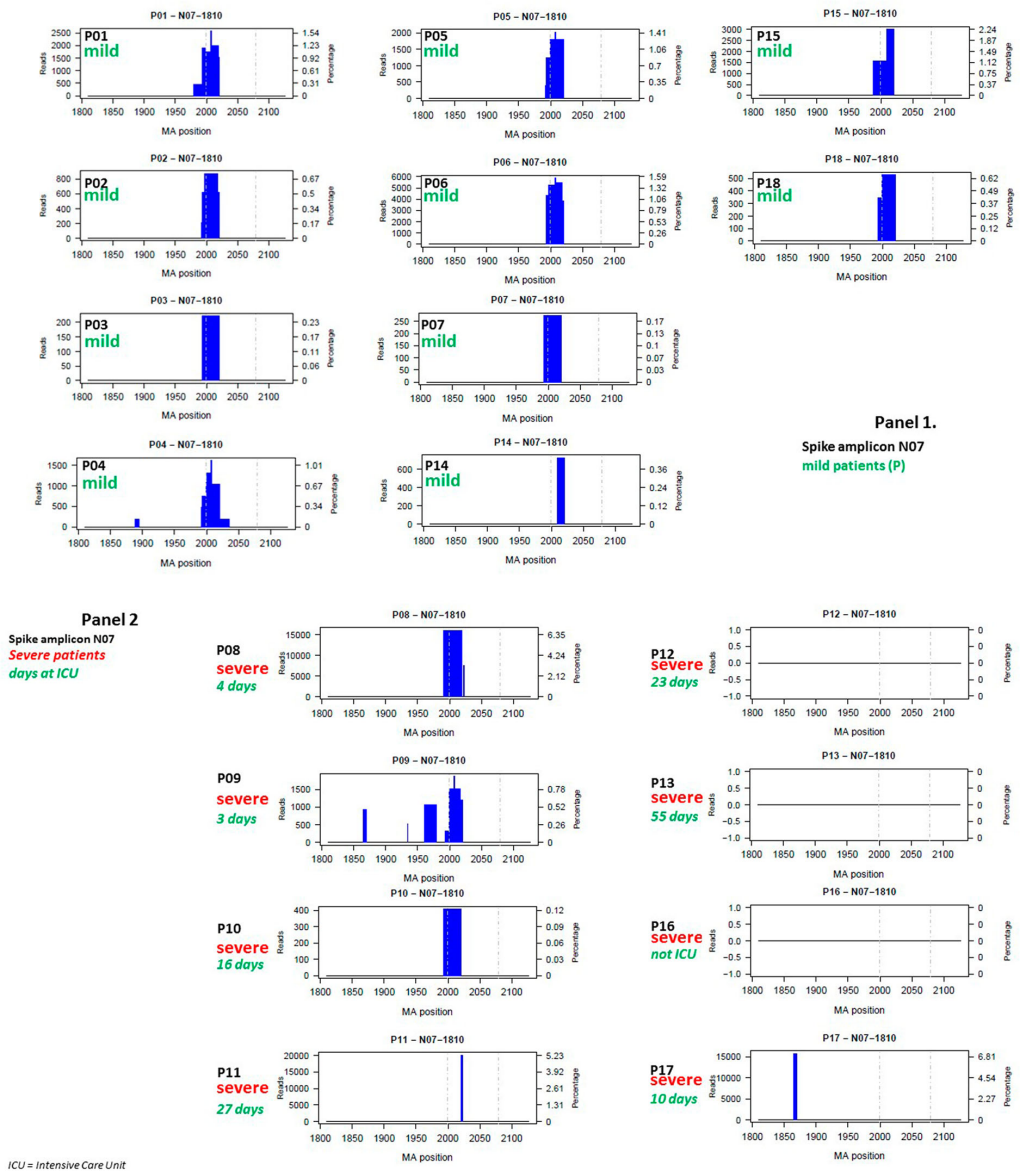
Bar plot of deletions in amplicon N07 in the 18 patients (P01-P18) at the nucleotide level: Panel 1, patients with mild disease; Panel 2, patients with severe disease. The x axis provides the multiple alignment (MA) nucleotide positions and the amplitude of the deletions by subregions, and the y axis shows the frequency of the deletion (percentage) on the right and the number of reads on the left. As no insertions were observed, the MA positions correspond to *S* gene positions. Dashed lines indicates S1/S2 (left) and S2’ (right) cleavage sites. Bar plots for the 18 patients by amplicons are provided in supplementary material (Figures S1 to S14 for nucleotides and S15 to S27 for amino acids).

A striking result was the accumulation of deletions (“hot-spot”) in amplicon N07, between nucleotides 1980–2035 (aa Y660-N679) in 14/18 (78%) patients, which included 100% of the patients with mild disease (P01-P07, P14, P15 and P18), and only half of those with severe disease (P08, P09, P10 and P11). In this particular hot-spot, deletions Δ2 to Δ34 were produced (Figure 2, Table 2). Among the severe patients, P12, P13, and P16 had no deletions in the N07 amplicon, and P17 showed a deletion outside this hot-spot location (Table 3). Viral variants carrying these deletions were significantly more frequent in mild than severe COVID-19 patients (Fisher test: odds-ratio: 95% confidence interval 0.0 - 0.9605; *p*=0.02288).

**Table 3.** List of deletions found in amplicon N07 at the nucleotide level aligned under the reference sequence Wuhan Hu-1 (MN908947.3). Alignment between nucleotides 1974 and 2070 is shown. Nucleotides represented in bold red in the reference sequence indicate the S1/S2 cleavage site R (CGT) / S (AGT).

Patient MILD/SEVERE	Nucleotide alignments
	
MN908947	**CTCATATGAGTGTGACATACCCATTGGTGCAGG**TATA **TGCGCTAGTTATCAGACTCAGACTAATTCTCCTCGGCGGGCACGT/AGTGTAGCTAGTCAA**
P01	CTCATATGAGTGTGACATACCCATTGGTGCAGG–TATGCGCTAGTTATCAGACTCAGACTAATTCTCCTCGGCGGGCACGT/AGTGTAGCTAGTCAA
P09	CTCATATGAGTGTGACATACCCATTGGTGCAGG–TATGCGCTAGTTATCAGACTCAGACTAATTCTCCTCGGCGGGCACGT/AGTGTAGCTAGTCAA
P05	CTCATATGAGTGTGACATACCCATTGGTGCAGG–TATGCGCTAGTTATCAGACTCAGACTAATTCTCCTCGGCGGGCACGT/AGTGTAGCTAGTCAA
P04	CTCATATGAGTGTGACATACCCATTGGTGCAGG–TATGCGCTAGTTATCAGACTCAGACTAATTCTCCTCGGCGGGCACGT/AGTGTAGCTAGTCAA
P01	CTCATATGAGTGTGACATACCCATT∼∼∼∼∼∼∼∼∼-TATGCGCTAGTTATCAGACTCAGACTAATTCTCCTCGGCGGGCACGT/AGTGTAGCTAGTCAA
P01	CTCATATGAGTGTGACATA∼∼∼∼∼∼∼∼∼∼∼∼∼∼∼-TATGCGCTAGTTATCAGACTCAGACTAATTCTCCTCGGCGGGCACGT/AGTGTAGCTAGTCAA
P04	CTCATATGAGTGTGACATA∼∼∼∼∼∼∼∼∼∼∼∼∼∼∼-TATGCGCTAGTTATCAGACTCAGACTAATTCTCCTCGGCGGGCACGT/AGTGTAGCTAGTCAA
P06	CTCATATGAGTGTGACATA∼∼∼∼∼∼∼∼∼∼∼∼∼∼∼-TATGCGCTAGTTATCAGACTCAGACTAATTCTCCTCGGCGGGCACGT/AGTGTAGCTAGTCAA
P09	CTCATATGAGTGTGACATA∼∼∼∼∼∼∼∼∼∼∼∼∼∼∼∼∼∼∼∼∼∼∼∼-GTTATCAGACTCAGACTAATTCTCCTCGGCGGGCACGT/AGTGTAGCTAGTCAA
P02	CTCATATGAGTGTGACATACCCA–∼∼∼∼∼∼∼∼∼∼∼∼∼∼∼∼∼∼–TTATCAGACTCAGACTAATTCTCCTCGGCGGGCACGT/AGTGTAGCTAGTCAA
P06	CTCATATGAGTGTGACATACCCATTGGTGCAGG-∼∼∼∼∼∼∼∼∼–TAATCAGACTCAGACTAATTCTCCTCGGCGGGCACGT/AGTGTAGCTAGTCAA
P06	CTCATATGAGTGTGACATACCCA–∼∼∼∼∼∼∼∼∼∼∼∼∼∼∼∼∼∼–TTATCAGACTCAGACTAATTCTCCTCGGCGGGCACGT/AGTGTAGCTAGTCAA
P08	CTCATATGAGTGTGACA–∼∼∼∼∼∼∼∼∼∼∼∼∼∼∼∼∼∼∼∼∼∼∼∼∼∼∼TATCAGACTCAGACTAATTCTCCTCGGCGGGCACGT/AGTGTAGCTAGTCAA
P08	CTCATATGAGTGTGACA–∼∼∼∼∼∼∼∼∼∼∼∼∼∼∼∼∼∼∼∼∼∼∼∼∼∼∼TATCAGACTCAGACTAATTCTCCTCGGCGGGCACGT/AGTGTAGCTAGTCAA
P08	CTCATATGAGTGTGACA–∼∼∼∼∼∼∼∼∼∼∼∼∼∼∼∼∼∼∼∼∼∼∼∼∼∼∼TATCAGACTCAGACTAATTCTCCTCGGCGGGCACGT/AGTGTAGCTAGTCAA
P01	CTCATATGAGTGTGACATACCCATTGGTGCAGG-∼∼∼∼∼∼∼∼∼∼∼∼TATCAGACTCAGACTAATTCTCCTCGGCGGGCACGT/AGTGTAGCTAGTCAA
P01	CTCATATGAGTGTGACATACCCATTGGTGCAGGTA–∼∼∼∼∼∼∼∼∼-ATCAGACTCAGACTAATTCTCCTCGGCGGGCACGT/AGTGTAGCTAGTCAA
P01	CTCATATGAGTGTGACATA∼∼∼∼∼∼∼∼∼∼∼∼∼∼∼∼∼∼∼∼∼∼∼∼∼∼∼-ATCAGACTCAGACTAATTCTCCTCGGCGGGCACGT/AGTGTAGCTAGTCAA
P02	CTCATATGAGTGTGACATA∼∼∼∼∼∼∼∼∼∼∼∼∼∼∼∼∼∼∼∼∼∼∼∼∼∼∼-ATCAGACTCAGACTAATTCTCCTCGGCGGGCACGT/AGTGTAGCTAGTCAA
P02	CTCATATGAGTGTGACAT-∼∼∼∼∼∼∼∼∼∼∼∼∼∼∼∼∼∼∼∼∼∼∼∼∼∼∼-ATCAGACTCAGACTAATTCTCCTCGGCGGGCACGT/AGTGTAGCTAGTCAA
P03	CTCATATGAGTGTGACATA∼∼∼∼∼∼∼∼∼∼∼∼∼∼∼∼∼∼∼∼∼∼∼∼∼∼∼-ATCAGACTCAGACTAATTCTCCTCGGCGGGCACGT/AGTGTAGCTAGTCAA
P04	CTCATATGAGTGTGACATACCCATTG–∼∼∼∼∼∼∼∼∼∼∼∼∼∼∼∼∼∼-ATCAGACTCAGACTAATTCTCCTCGGCGGGCACGT/AGTGTAGCTAGTCAA
P04	CTCATATGAGTGTGACATACCCATT∼∼∼∼∼∼∼∼∼∼∼∼∼∼∼∼∼∼∼∼∼-ATCAGACTCAGACTAATTCTCCTCGGCGGGCACGT/AGTGTAGCTAGTCAA
P04	CTCATATGAGTGTGACAT-∼∼∼∼∼∼∼∼∼∼∼∼∼∼∼∼∼∼∼∼∼∼∼∼∼∼∼-ATCAGACTCAGACTAATTCTCCTCGGCGGGCACGT/AGTGTAGCTAGTCAA
P05	CTCATATGAGTGTGACATACCCATT∼∼∼∼∼∼∼∼∼∼∼∼∼∼∼∼∼∼∼∼∼-ATCAGACTCAGACTAATTCTCCTCGGCGGGCACGT/AGTGTAGCTAGTCAA
P05	CTCATATGAGTGTGACATA∼∼∼∼∼∼∼∼∼∼∼∼∼∼∼∼∼∼∼∼∼∼∼∼∼∼∼-ATCAGACTCAGACTAATTCTCCTCGGCGGGCACGT/AGTGTAGCTAGTCAA
P05	CTCATATGAGTGTGACAT-∼∼∼∼∼∼∼∼∼∼∼∼∼∼∼∼∼∼∼∼∼∼∼∼∼∼∼-ATCAGACTCAGACTAATTCTCCTCGGCGGGCACGT/AGTGTAGCTAGTCAA
P06	CTCATATGAGTGTGACATA∼∼∼∼∼∼∼∼∼∼∼∼∼∼∼∼∼∼∼∼∼∼∼∼∼∼∼-ATCAGACTCAGACTAATTCTCCTCGGCGGGCACGT/AGTGTAGCTAGTCAA
P07	CTCATATGAGTGTGACATA∼∼∼∼∼∼∼∼∼∼∼∼∼∼∼∼∼∼∼∼∼∼∼∼∼∼∼-ATCAGACTCAGACTAATTCTCCTCGGCGGGCACGT/AGTGTAGCTAGTCAA
P09	CTCATATGAGTGTGACATACCCATT∼∼∼∼∼∼∼∼∼∼∼∼∼∼∼∼∼∼∼∼∼-ATCAGACTCAGACTAATTCTCCTCGGCGGGCACGT/AGTGTAGCTAGTCAA
P10	CTCATATGAGTGTGACATA∼∼∼∼∼∼∼∼∼∼∼∼∼∼∼∼∼∼∼∼∼∼∼∼∼∼∼-ATCAGACTCAGACTAATTCTCCTCGGCGGGCACGT/AGTGTAGCTAGTCAA
P14	CTCATATGAGTGTGACATACCCATTGGTGCAGGTA–∼∼∼∼∼∼∼∼∼-ATCAGACTCAGACTAATTCTCCTCGGCGGGCACGT/AGTGTAGCTAGTCAA
P15	CTCATATGAGTGTGACATACCCATTGGTGCAGGTA–∼∼∼∼∼∼∼∼∼-ATCAGACTCAGACTAATTCTCCTCGGCGGGCACGT/AGTGTAGCTAGTCAA
P18	CTCATATGAGTGTGACATA∼∼∼∼∼∼∼∼∼∼∼∼∼∼∼∼∼∼∼∼∼∼∼∼∼∼∼-ATCAGACTCAGACTAATTCTCCTCGGCGGGCACGT/AGTGTAGCTAGTCAA
P18	CTCATATGAGTGTGACATACCCATT∼∼∼∼∼∼∼∼∼∼∼∼∼∼∼∼∼∼∼∼∼-ATCAGACTCAGACTAATTCTCCTCGGCGGGCACGT/AGTGTAGCTAGTCAA
P06	CTCATATGAGTGTGACATA∼∼∼∼∼∼∼∼∼∼∼∼∼∼∼∼∼∼∼∼∼∼∼∼∼∼∼–TCAGACTCAGACTAATTCTCCTCGGCGGGCACGT/AGTGTAGCTAGTCAA
P09	CTCATATGAGTGTGACATACCCATT∼∼∼∼∼∼∼∼∼∼∼∼∼∼∼∼∼∼∼∼∼–TCAGACTCAGACTAATTCTCCTCGGCGGGCACGT/AGTGTAGCTAGTCAA
P15	CTCATATGAGTGTG–∼∼∼∼∼∼∼∼∼∼∼∼∼∼∼∼∼∼∼∼∼∼∼∼∼∼∼∼∼∼–TCAGACTCAGACTAATTCTCCTCGGCGGGCACGT/AGTGTAGCTAGTCAA
P01	CTCATA-∼∼∼∼∼∼∼∼∼∼∼∼∼∼∼∼∼∼GGTGCAGG **TATA**TGCGCTAGTTATCAGACTCAGACTAATTCTCCTCGGCGGGCACGT/AGTGTAGCTAGTCAA
P08	CTCATATGAGTGTGACATACCCATTGGTGCAGG **TATA**TGCGCTAGTTA–AGACTCAGACTAATTCTCCTCGGCGGGCACGT/AGTGTAGCTAGTCAA
P11	CTCATATGAGTGTGACATACCCATTGGTGCAGG **TATA**TGCGCTAGTT—AGACTCAGACTAATTCTCCTCGGCGGGCACGT/AGTGTAGCTAGTCAA
P11	CTCATATGAGTGTGACATACCCATTGGTGCAGG **TATA**TGCGCTAGTT—AGACTCAGACTAATTCTCCTCGGCGGGCACGT/AGTGTAGCTAGTCAA
P04	CTCATATGAGTGTGACATACCCATTGGTGCAGG **TATA**TGCGCTAGTT–∼∼∼∼∼∼∼∼∼∼∼∼-ATTCTCCTCGGCGGGCACGT/AGTGTAGCTAGTCAA

Among the total of 43 deletions detected in amplicon N07 (Table 3), a premature stop codon appeared immediately after the deletion site in 5 cases (11.6%) and the reading frame recovered after losing 4, 5, or 7 amino acids in 6 cases. However, a frameshift that changed the reading frame and caused the appearance of a premature stop codon several amino acids later was generated in most of the deletions 32/43 (74.4%), and in consequence the S1/S2 cleavage site and the polybasic domain (PRRAR/S) disappeared. In 39 of the 43 (90.7%) N07 deletions, a TATA box-like motif (nt 2,007–2,010) was lost. In this particular region, the deletion was characterized by a similar 3’ cutting edge (Table S11). An interesting result at the amino acid level was that regardless of the starting point of the deletion (nt 654, 663, 664, 665, 666, 667 or 671), in 9 of the mild patients (all except P14) and in 2 severe ones (P09 and P10), the frameshift caused by the deletion generated a new peptide motif, IRLRLILLGGHVV*, with a stop codon (*) at the end (Table 4).

**Table 4.** Deletions in amplicon N07 at the amino acid (aa) level. wt = wild type (MN908947.3). S, stop; Lost + S, loss of reading frame and appearance of a stop codon; rRF, recover reading frame. Haplotypes that did not lose the TATA box-like sequence are highlighted in yellow, and haplotypes with a deletion upstream of the TATA box-like sequence are highlighted in blue. Cleavage S1/S2 amino acid site between residues 685 / 686 (PRRAR/S). * stop codon.

Patient MILD/SEVERE	Amino acid alignments	
MN908947.3	**5'654 EHVNNSYECDIPIGAGICASYQTQTNSPRRAR/SVASQSIIAYTMSLGAENSVAYS 708 3'**	*** ***
P01	EHVNNSYECDIPIGAGMR*	S
P09	EHVNNSYECDIPIGAGMR*	Lost + S
P05	EHVNNSYECDIPIGAGMR*	Lost + S
P04	EHVNNSYECDIPIGAGMR*	Lost + S
P01	EHVNNSYECDIPIYALVIRLRLILLGGHVV*	Lost + S
P01	EHVNNSYECDIYALVIRLRLILLGGHVV*	Lost + S
P04	EHVNNSYECDIYALVIRLRLILLGGHVV*	Lost + S
P06	EHVNNSYECDIYALVIRLRLILLGGHVV*	Lost + S
P09	EHVNNSYECDIVIRLRLILLGGHVV*	Lost + S
P02	EHVNNSYECDIPIIRLRLILLGGHVV*	Lost + S
P06	EHVNNSYECDIPIGAG—NQTQTNSPRRAR/SVASQSIIAYTMSLGAENSVAYS	rRF
P06	EHVNNSYECDIPIIRLRLILLGGHVV*	Lost + S
P08	EHVNNSYECDISDSD*	Lost + S
P08	EHVNNSYECDISDSD*	Lost + S
P08	EHVNNSYECDISDSD*	Lost + S
P01	EHVNNSYECDIPIGAGIRLRLILLGGHVV*	Lost + S
P01	EHVNNSYECDIPIGAG———NQTQTNSPRRAR/SVASQSIIAYTMSLGAENSVAYS	rRF
P01	EHVNNSYECDIIRLRLILLGGHVV*	Lost + S
P02	EHVNNSYECDIIRLRLILLGGHVV*	Lost + S
P02	EHVNNSYECDISDSD*	Lost + S
P03	EHVNNSYECDIIRLRLILLGGHVV*	Lost + S
P04	EHVNNSYECDIPI——-DQTQTNSPRRAR/SVASQSIIAYTMSLGAENSVAYS	rRF
P04	EHVNNSYECDIPIIRLRLILLGGHVV*	Lost + S
P04	EHVNNSYECDISDSD*	Lost + S
P05	EHVNNSYECDIPIIRLRLILLGGHVV*	Lost + S
P05	EHVNNSYECDIIRLRLILLGGHVV*	Lost + S
P05	EHVNNSYECDISDSD*	Lost + S
P06	EHVNNSYECDIIRLRLILLGGHVV*	Lost + S
P07	EHVNNSYECDIIRLRLILLGGHVV*	Lost + S
P09	EHVNNSYECDIPIIRLRLILLGGHVV*	Lost + S
P10	EHVNNSYECDIIRLRLILLGGHVV*	Lost + S
P14	EHVNNSYECDIPIGAG—-NQTQTNSPRRAR/SVASQSIIAYTMSLGAENSVAYS	rRF
P15	EHVNNSYECDIPIGAG—-NQTQTNSPRRAR/SVASQSIIAYTMSLGAENSVAYS	rRF
P18	EHVNNSYECDIIRLRLILLGGHVV*	Lost + S
P18	EHVNNSYECDIPIIRLRLILLGGHVV*	Lost + S
P06	EHVNNSYECDISDSD*	Lost + S
P09	EHVNNSYECDIPISDSD*	Lost + S
P15	EHVNNSYECVRLRLILLGGHVV*	Lost + S
P01	EHVNNS*	S
P08	EHVNNSYECDIPIGAGICAS*	S
P11	EHVNNSYECDIPIGAGICAS*	S
P11	EHVNNSYECDIPIGAGICAS*	S
P04	EHVNNSYECDIPIGAGICASY **—-**SPRRAR/SVASQSIIAYTMSLGAENSVAYS	rRF

In 9 patients, a second deletion hot-spot was found deleting a number of nucleotides (from 2 to 16) between positions 2451 and 2467 (aa 817F-823F), coinciding with the secondary S cleavage site (S2’). The hot spot was located between nt2431 (811K) and nt2454 (818I), just after the exact S2’ cleavage site (KPSKR/SFI) (Table 2).

## Discussion

Here, we describe the naturally occurring deletions in the SARS-CoV-2 *S* gene in a set of patients with mild or severe COVID 19. The deletions mainly clustered in two hot-spot regions, one (Δ15, affecting aa660-aa679) located upstream but very close to the S1/S2 cleavage site (aa 685/686) and the second (Δ16 affecting aa817-823) situated just upstream to the secondary cleavage site S2’ (aa 815/816). These two deletions were found in most of the patient samples studied, and notably, the Δ15 deletion was present in 100% of patients with mild infection and in half of those with severe disease, three quarters of the patients studied (Table 2). This finding suggests that the deletions are not sporadic events even though they were seen in a relatively small percentage of the viral quasispecies (2.2% for Δ15; and 0.54% for Δ16). The mutants could be interpreted as a strategy that natural selection has favoured during the SARS-CoV-2 infectious life cycle to facilitate extensive spread of the infection, as is discussed below.

This study involved deep-sequencing of the complete SARS-CoV-2 *spike* gene using 13 overlapping amplicons in laboratory-confirmed samples for SARS-CoV-2 in 18 patients. In studies with other SARS-CoV viruses, several subgenomic RNAs were reported to be generated during the cell cycle [6,18]. To exclusively study the genomic viral RNA of SARS-CoV-2, RT–PCR was performed using two large PCR products in which the 5’ end of primer pair 1 and the 3’ end of primer pair 2 were designed to be outside the spike region (5’ end in ORF1ab and 3’ end in ORF3a) (Table S11, Figure S14). Taking into consideration that CoV have 3’-5’ ExoN activity (nsp14 protein), consistent with a proofreading mechanism to correct mutations during replication, the deep-sequencing analysis accepted mutants present at a low frequency of ≥0.1%. Because of the possibility of PCR artefacts, deep-sequencing point mutations, or deletion of single nucleotides generated mainly at homopolymeric sites, we did not include single deletions unless they were found in different patients and in overlapping amplicons at higher frequencies (>1%). No insertions were found.

**Figure 3.**
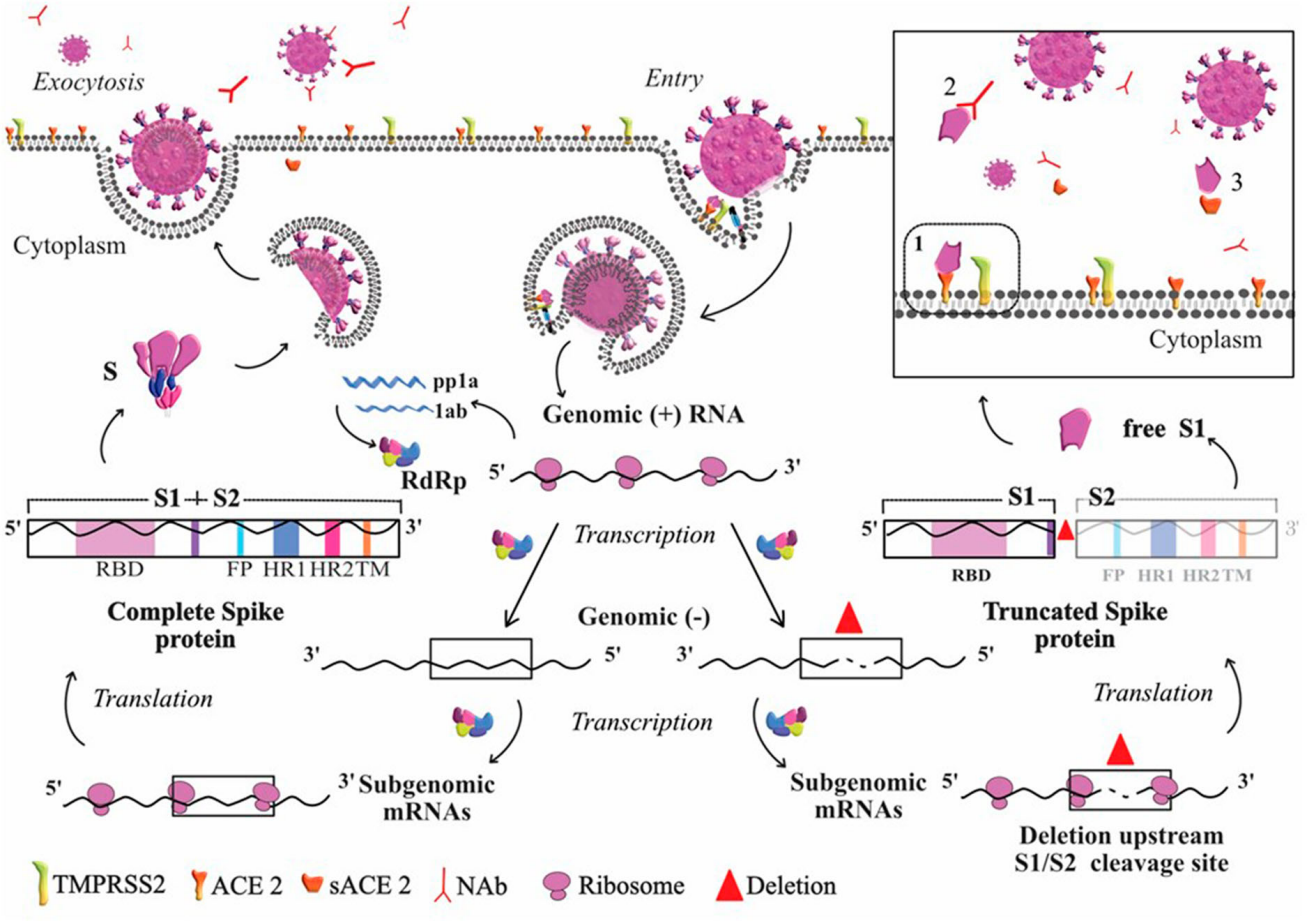
Based on the life cycle of SARS-CoV, this diagram represents the hypothesis derived from our results. Entry of the virus in the host cell is shown at the top right of the diagram. At the transcription step, two scenarios are depicted: to the left, the viral particle resulting from normal S protein, and to the right the viral particle resulting from truncated S protein. In normal conditions, once the nucleoprotein is freed into the cytoplasm ss + RNA is translated into the non-structural proteins required for transcription. ss + RNA is transcribed into ss-RNA and later into genomic ss + RNA which is encapsidated (left side of the figure). Once the complete viral particle has been formed, it is secreted from the cell by exocytosis. The right side of the figure depicts the situation when a deletion occurs in the *S* gene during transcription of the complete genome and before subgenomic mRNAs are generated to produce the structural proteins. Translation of a deleted subgenomic spike mRNA would lead to a truncated S protein composed of the S1 domain without S2, which could be shed outside the cell as free S1. The box depicts possible destinations of free S1, which could bind to (1) the ACE2 cell receptor, (2) S1-specific neutralizing antibodies, or (3) free ACE2 receptor. ***The red triangle indicates the deletion in genomic RNA. ***Abbreviations: ACE2, angiotensin converting enzyme 2; mRNA, messenger RNA; NAb; neutralizing antibodies; pp1a, polyprotein 1a; RdRp, RNA-dependent RNA polymerase; S, spike; S1, subunit S1 at the N-terminal domain of the S protein, which includes receptor binding domain (RBD); S2, subunit S2 located at the C-terminal domain of S protein, which includes fusion peptide (FP), heptad repeat (HR) domain 1 and 2, and the transmembrane domain (TM); ss, single stranded; ss + RNA, single-stranded positive sense RNA; TMPRS22, human serine protease TMPRSS2.

Entry of the viral genome into the cell depends on recognition and binding of the surface subunit S1 to the ACE2 human receptor [11], whereas the S2 subunit is responsible for fixing the S protein to the viral membrane surface. After binding to the ACE2 cell receptor, the S protein is primed by the serin-protease TMPRSS2, which leads to S protein cleavage at S1/S2 and S2’ [8]. After cleavage, S1 remains attached to ACE2, while subunit S2 anchors the viral and cellular membranes, inducing fusion and viral entry. The Δ15 deletion (Table 2) mainly causes a frameshift that generates an in-frame stop codon. The presence of this new stop codon would result in translation of a truncated S, which would consist of an almost complete S1 subunit, and total absence of the S2 subunit responsible for anchoring S to the lipid membrane of the viral particle. The absence of the S2 anchor peptide suggests that S1 could be produced as a “free” protein (free S1). As S1 is located on the exposed outside of SARS-CoV-2 in the crown structures, it could have hydrophilic domains and be a soluble peptide with potential for release outside the infected cell, in the lower respiratory tract and even to plasma (Figure 3). These free soluble proteins, which are not a part of the viral cycle or components of the viral particles have also been observed in other viral infections. For example, a huge amount of “empty” subviral genomic particles, consisting of viral envelope proteins (HBsAg), are often found in plasma of patients with hepatitis B virus (HBV) infection. These empty particles are produced and secreted during HBV infection, and have an immunomodulatory role [19]. In addition, soluble HBV e antigen (HBeAg), which is not a component of the viral particles and shares immunoactive epitopes with the HBV core antigen (HBcAg viral capsid component), is detected during HBV infection and has an immunomodulatory role [20].

Human respiratory syncytial virus (HRSV) is another respiratory virus with the ability to produce pre-anchored proteins. The attachment protein (G) of HRSV is an anchored protein whose main function is viral attachment to the host’s cell membrane through a still unknown receptor [21]. As in many other viruses, this protein has several functions, and in this case, because of the existence of a second start codon, a soluble form of G protein lacking the anchor is produced, and this is shed to the extracellular medium [22] in abundant quantities by infected cells. The function of soluble, free G is to inhibit toll-like receptors, thereby modulating the host’s immune response. Free G also binds to the host’s neutralizing antibodies, which are mainly directed to this protein. In this way, neutralization of circulating virions is reduced, favouring viral infection [23].

The free S1 binding subunit of SARS-CoV-2 without its membrane anchor S2 could have similar functions (Figure 3). One putative action of secreted free S1 protein might be to attach to the human ACE2 cell receptor, thereby competing with complete viral particles to re-infect or newly infect respiratory tract cells, resulting in less severe disease. This could be interpreted as an effect of natural selection to attenuate the infection and facilitate its persistence with minimal damage, increasing the human-to-human transmission into the community. This strategy, which we have dubbed “Don’t burn down the house” is supported by the finding that the minor variants carrying these deletions were statistically more frequent in patients with mild than severe COVID-19. This self-modulating viral strategy has also been seen in hepatitis delta virus (HDV) infection, where one viral antigen (short HDV antigen, SHDAg) enhances HDV replication, while a second antigen (large HDV antigen, LHDAg), produced after a stop codon edition (TAG to TGG) by cellular adenosine deaminase, acts as a negative regulator of replication [24].

The fact that the truncated S protein was present in only a low percentage of the entire viral quasispecies suggests that natural selection may have designed a favourable equilibrium in which a limited number of deleted virions are generated to balance virus production with infection of new cells during disease progression. A likely reason for maintaining a minority population of genomes with deletions able to produce free S1 protein would be to infect a host while causing minimal damage, which would greatly facilitate transmission of the virus within the population. However, the mutants were also found in half the patients with severe disease; hence, additional study is needed to determine whether they also relate to disease severity. In clinical practice, it has been seen that progression to severe disease can occur within hours, which suggests that any variant associated with virulence should be detected at the time of the diagnosis. The samples studied here were obtained on the day patients were admitted to the emergency room, and very close to the onset of infection. Does the percentage of these viral mutants change during disease progression? To elucidate this issue, it would be of interest to investigate changes in the frequency of deleted genomes in a large number of patients and in sequential samples from the same patient, together with virus culture experiments to determine whether the presence of deleted sequences increases or not during the passages. As a consequence of the frameshift, a new peptide motif, IRLRLILLGGHVV*, appeared in several sequences with a deletion that started in different nucleotide points. Additional work is also needed to determine whether acquisition of this peptide motif has biological consequences.

Two other putative consequences of the *S* mutants might be that free S1 protein could bind with S-specific antibodies, acting as a decoy and weakening the immune response, or to circulating ACE2, released from the cell membrane to plasma [25,26], with cardiovascular effects. However, as the deletions were mainly found in patients with mild disease and considering the zoonotic origin of the virus (animal immune and cardiac systems differ from human ones) and the short time that the virus has been evolving in the human population, we believe that the most likely reason for maintaining a minor population of mutant genomes able to produce free S1 protein would be to cause an infection with limited damage in the host, thus facilitating transmission and persistence of the virus in the population. The observation of mutation hot spots in the *S* gene opens the door to further work on a number of potentially related aspects.

Recent studies have reported the presence of deleted variants in the S1/S2 junction in virus isolated by cell culture of clinical specimens [27]. Deletions of 10–15 nucleotides at the S1/S2 junction were identified by plaque purification of Vero-E6 cultured SARS-CoV-2 genomes obtained from nasopharyngeal aspirate of a COVID-19 patient. Infection of hamsters with virus containing these variants led to attenuated viral disease [27,28]. Digital PCR-based assays demonstrated that such mutants carrying deletions at low intra-host frequency can also be transmitted from human to human, which suggests that they may have significant implications in the zoonotic origin and natural evolution of SARS-CoV-2 [28]. These findings support our hypothesis that deletions close to the S1/S2 cleavage site are likely a natural phenomenon. Here, we suggest that this phenomenon may have been favoured by natural selection to enhance the spread of SARS-CoV-2.

To conclude, in-depth sequencing of the SARS-CoV-2 *S* gene in 18 patients with COVID-19 enabled identification of a naturally occurring deletion very close to the S1/S2 cleavage site. Our results indicate that the mutant S would have a large impact on the S protein, and suggest that the virus could produce free S1, which may have implications regarding the candidacy of S protein as a target for vaccination and antiviral treatment strategies. The deletions were significantly more prevalent in patients with mild than in those with severe disease, supporting the notion that they could be a strategy of natural selection to decrease the injury caused after onset of the infection. In this “Don’t burn down the house” strategy, the ability of the virus to bind with ACE2 receptor and spread to others would be unchanged; thus its propensity for transmission would be enhanced by a mildly affected host. To prove this hypothesis, it is essential to investigate whether the truncated S protein (free S1) is present in respiratory tract specimens and in plasma. To detect free S1 at low concentration by western blot analysis, entire and truncated recombinant spike proteins should be used as controls, together with highly specific antibodies to S protein. These studies are currently ongoing, and in parallel, we are investigating whether the new peptide motif IRLRLILLGGHVV* will have sufficient antigenicity to be used as a probe to detect truncated free S1 protein.
